# Involvement and continuity in “Initiation, Evolution, Excellency”

**Published:** 2013-12-25

**Authors:** VL Purcarea

**Affiliations:** Carol Davila University of Medicine and PharmacyRomania

The Days of “Carol Davila” University of Medicine and Pharmacy in Bucharest have become a mark of pride, happiness and balance for the biggest and oldest Romanian school of medicine.

**Fig. 1:**
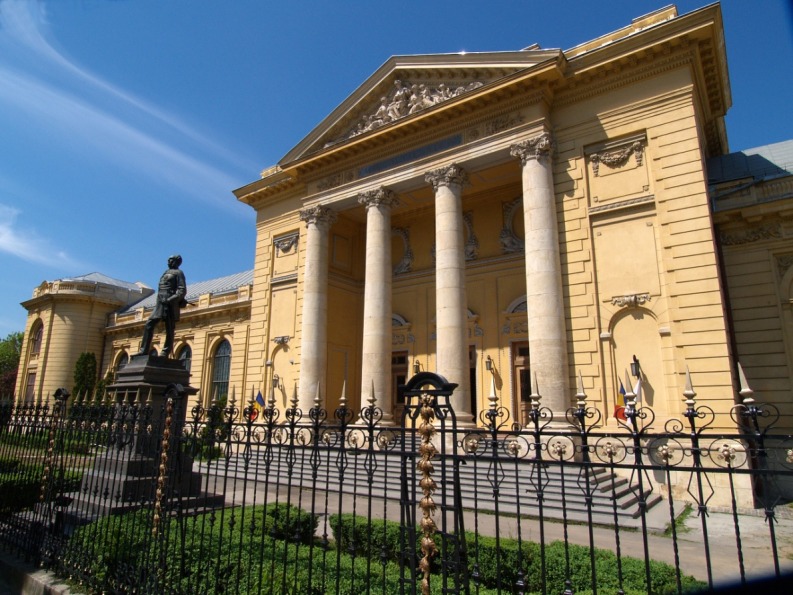
The main building of “Carol Davila” University of Medicine and Pharmacy

The official opening of the University Days has taken place in “George Emil Palade” imposing amphitheater of “Carol Davila” University of Medicine and Pharmacy, which was packed with professors, students and parents, in the vibrant sound of the traditional “Gaudeamus” – “let us be happy as long as we are still young”- the members of “VOX Medicalis” chorus of students of “Carol Davila” University of Medicine and Pharmacy in Bucharest have sang with talent and abnegation, inciting all the people gathered in the Aula Magna to join them, in such a manner that the final lines “long live the school and the professors may they flourish forever” have been sang by everybody, generating a high intensity emotional state.

**Fig. 2:**
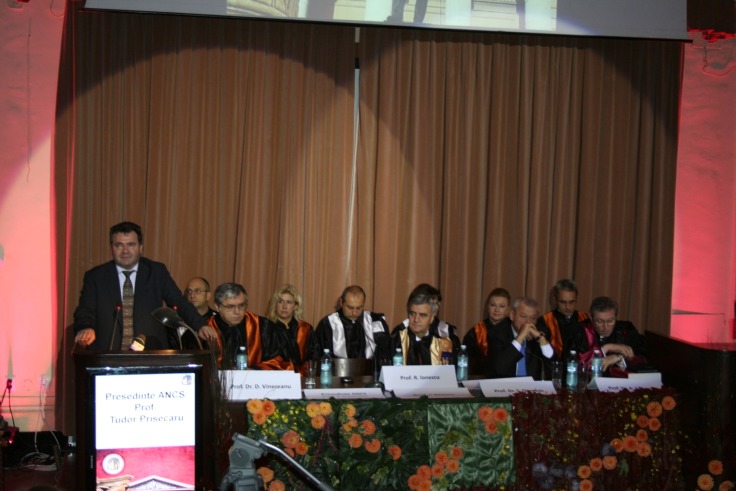
The University Leadership and the guests of honor – The Days of “Carol Davila” University of Medicine and Pharmacy

Simultaneously with the Official Opening of the University Year 2013-2014 and the “University Days - the 2013 edition”, the Rector of the University, Academician Ioanel Sinescu, has addressed in his speech with priority to the new colleagues in the 1st year, sharing some reflections of the ancestors regarding the chosen occupation “for which you have to continuously learn and never learn enough”, “an occupation in which there is no excuse for improvisation”, and, a wonderful occupation which can make people happy, an occupation without limits, “in which mediocrity and incompetence are prohibited!”, urging, through these reflections, to a conscious and responsible effort of professional forming, of integration in the medical school in Bucharest, a generous space of the professional performance, of intellectual solidarity and of fair-play competency, a true edifice of efforts, work, accomplishments and professional performances. The same warm and sincere thoughts have been transmitted to the 1st year students and, of course, not only to them, both by the President of the University Senate, Prof. Adrian Streinu Cercel, MD., but also by other remarkable members of the Administration Council of “Carol Davila” University of Medicine and Pharmacy. Among the guests of honor of the event there have been Prof. Tudor Prisecaru, MD, ANCS president as well as The General Mayor of Bucharest, our colleague, Prof. Sorin Oprescu, MD, who, with wisdom and sensitivity have shared glimpses of their rich university activity and have addressed special success wishes which came from their hearts, many accomplishments and a beautiful, efficient and full of joy period during their student years. The University Days have continued with the “Opening of the National Saloon of University Book – the IXth edition”, “The official opening of the Students of “Carol Davila” University of Medicine and Pharmacy, the “Official Opening of the University Library Day – the XVIth edition and “have continued the next day with manifestations dedicated to the celebration of **„90 years of pharmaceutical individual education” in the Faculty of Pharmacy and the Action of Oath Submission of the series of graduates 2013. **

On this occasion, the following exhibitions have been organized:** “Personalities of “Carol Davila” University of Medicine and Pharmacy”; “Models of dedication and devotion – the Rectors gallery”; “University Book – “The present of the past – continuity in valuable medical papers of Medicine in Bucharest””; “Rare medical books and atlases”, and “Bucharest Medicine Students – ideas and projects”** Projects Fair has taken place in the same period of time.

**Fig. 3:**
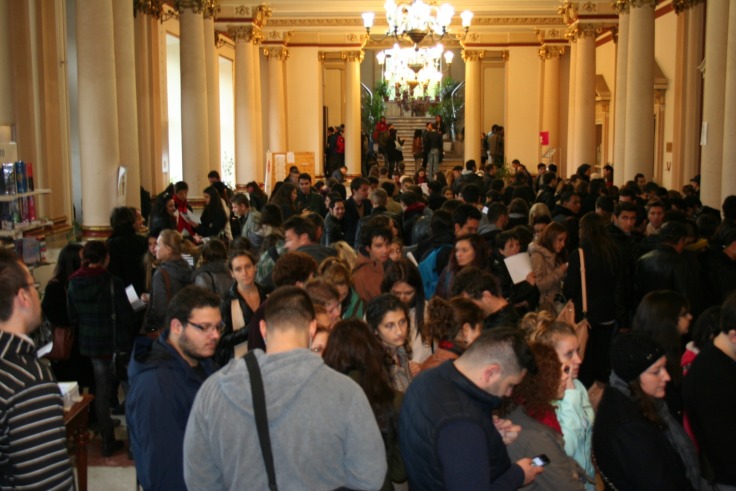
The students’ Projects Fair – The Days of Carol Davila University of Medicine and Pharmacy 2013

During the event, many attractive **“Students sports competitions – The series of students who are the most keen on sports!”**, such as in swimming, basketball, volleyball, tennis and football, have taken place. “The University Days” have ended with the awarding ceremony of the most important accomplishments and of the most appreciated participants, a ceremony which has taken place in the impressive Council Hall of the University. **“Carol Davila” Diploma** has been awarded to: Prof. Catalin Cirstoiu, MD, for the following book titles: “Osteoporosis in menopause”, “The infection of joint implants” and “The Clinical examination in orthopedics”, books which were launched in the “Interdisciplinary perspectives” International Congress, which took place in the Palace of Parliament.

**Fig. 4:**
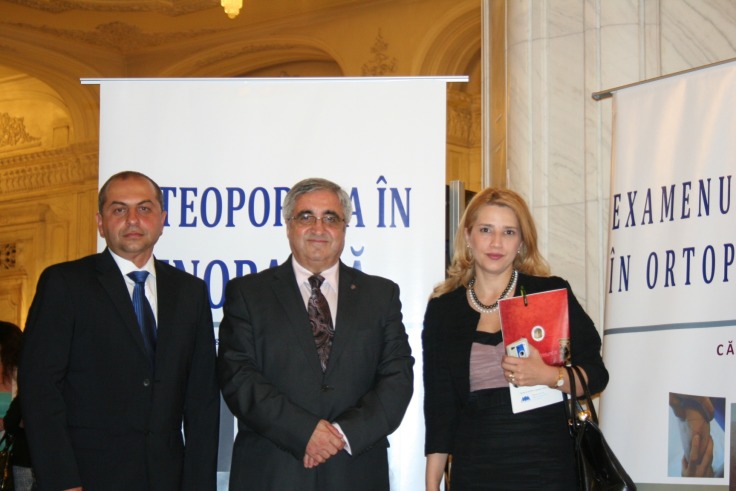
“Carol Davila” University of Medicine and Pharmacy Congress 2013

“Carol Davila” Diploma has also been awarded to Prof. Doina Plesca, MD, and Prof. Mihaela Balgradean, MD, for the following book titles: “Nutritional recommendations in pediatric practice” and “Severe infection in pediatric pathology”.

**Fig. 5:**
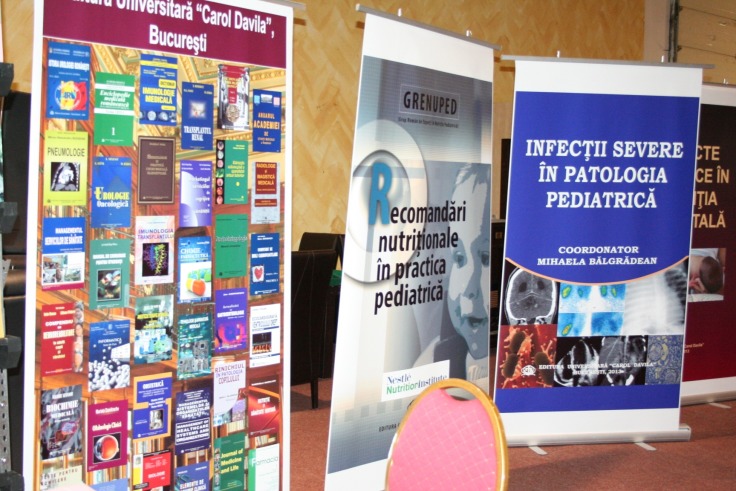
The Congress of Pediatrics – Tg. Mures, Romania

**Fig. 6:**
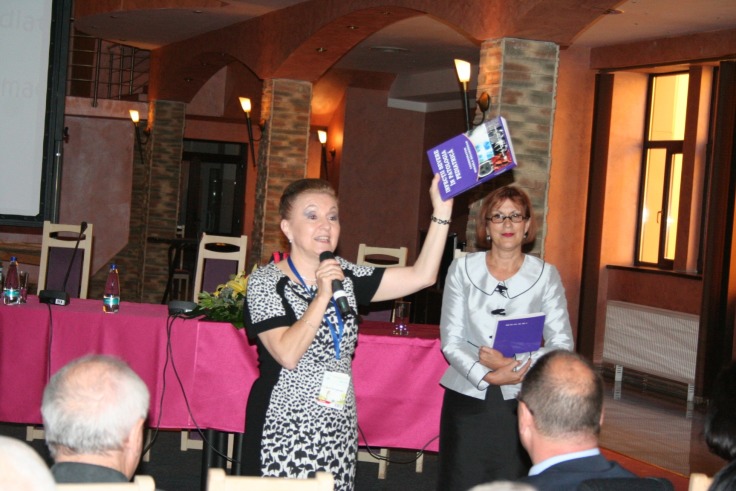
Books launched in the National Congress of Pediatrics – Tg. Mures, Romania

A book which was launched in the Second National Conference with International Participation of the Romanian Society of Hypertension, in Bucharest is the following: Prof. Mioara Dorobantu, MD – “Point and counterpoint in arterial hypertension”.

**Fig. 7:**
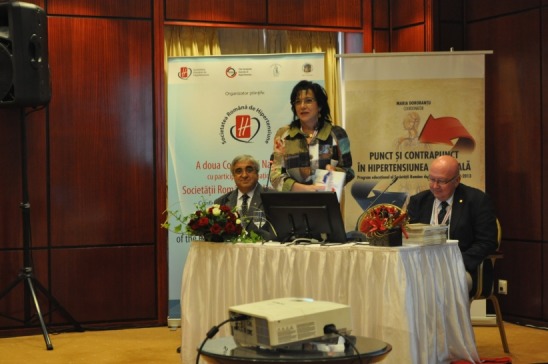
Second National Conference with International Participation of the Romanian Society of Hypertension

Moreover, Prof. Silvia Stoicescu, MD, has also launched the book “Practical aspects in neo-natal nutrition”, which was first launched in the XVIth National Conference of Neonatology.

**Fig. 8:**
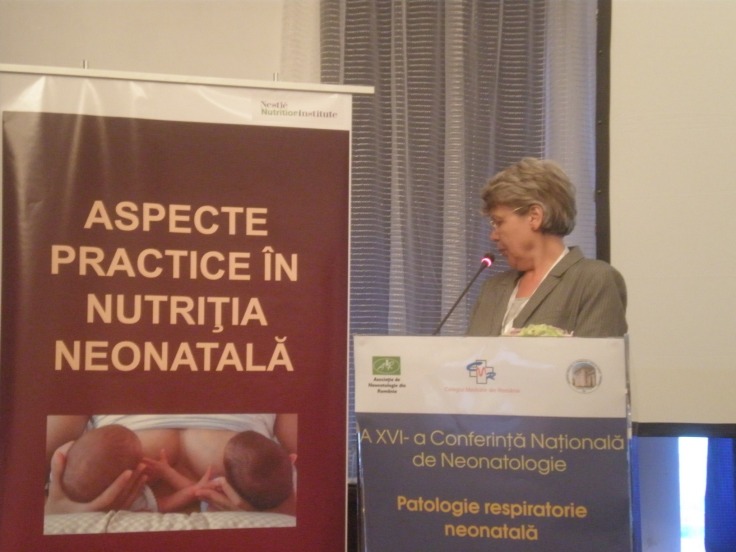
The National Conference of Neonatology – Murighiol

**Fig. 9:**
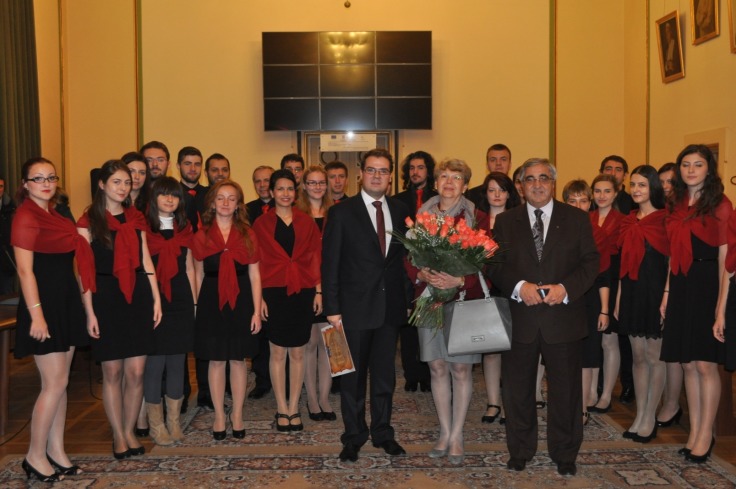
“Carol Davila” University Days 2013 – Awards Ceremony

**The Diploma “The University Book Saloon”** has been awarded to the following book titles: “Endourology treaty” author Prof. Petrisor Geavlete, MD; “The kidney in systemic lupus erythematosus” author M. Penescu; “Descriptive inorganic chemistry” authors Nacea Veronica and Rica Boscenco; “Diagnosis and treatment guide for hepatic virus infection” author Ileana Constantinescu; “Thermal treatment of bone tumors” author Dan Noveanu; “Behavioral sciences in medicine” author Ovidiu Popa Velea; “The essential in chronic renal insufficiency” author Checherita Ionel; “Fostering innovation in heartcare services” authors Th. Borangiu and Radu Dobrescu; “Ligand metal complexes which contain the azometric ligand – new perspectives in the modulation of coordination geometry” author E.M. Pahontu and the album “Neurophysiologic opera of shopping” by Bogdan Lascar. 

After the Awards Ceremony, “Vox Medicalis” Chorus has given the colleagues and professors a wonderful representation, which was considered a quasi-educative aspect, having explanations regarding the intentions of the composers, an incursion in the music analysis of the songs and the disclosure of some little secrets from the rehearsals laboratory, a recital which was cheered for many minutes, in “George Emil Palade” Amphitheater of “Carol Davila” University of Medicine and Pharmacy, Bucharest. 

